# Relationship between wintering site and survival in a migratory waterbird using different migration routes

**DOI:** 10.1007/s00442-024-05518-x

**Published:** 2024-02-24

**Authors:** Hugo R. S. Ferreira, Jocelyn Champagnon, José A. Alves, Tamar Lok

**Affiliations:** 1 Department of Biology & CESAM—Centre for Environmental and Marine Studies, University of Aveiro, Campus de Santiago, 3810-193 Aveiro, Portugal; 2 Tour du Valat, Research Institute for the Conservation of Mediterranean Wetlands, Le Sambuc, 13200 Arles, France; 3 South Iceland Research Centre, University of Iceland, Lindarbraut 4, 840-IS Laugarvatn, Iceland; 4 Department of Coastal Systems, NIOZ Royal Netherlands Institute for Sea Research, Texel, The Netherlands

**Keywords:** Apparent survival, Capture-recapture, Cormack–Jolly–Seber models, Life history, *Platalea leucorodia*, Pre-breeding migration, Resighting probability, Seasonal migration, Wintering strategies

## Abstract

When wintering at different sites, individuals from the same breeding population can experience different conditions, with costs and benefits that may have implications throughout their lifetime. Using a dataset from a longitudinal study on Eurasian Spoonbills from southern France, we explored whether survival rate varied among individuals using different wintering sites. In the last 13 years, more than 3000 spoonbills have been ringed as chicks in Camargue. These birds winter in five main regions that vary in both migratory flyway (East Atlantic vs. Central European) and migration distance (long-distance vs. short-distance vs. resident). We applied Cormack–Jolly–Seber models and found evidence for apparent survival to correlate with migration distance, but not with flyway. During the interval between the first winter sighting and the next breeding period, long-distance migrants had the lowest survival, independently of the flyway taken. Additionally, as they age, spoonbills seem to better cope with migratory challenges and wintering conditions as no differences in apparent survival among wintering strategies were detected during subsequent years. As dispersal to other breeding colonies was rarely observed, the lower apparent survival during this period is likely to be partly driven by lower true survival. This supports the potential role of crossing of natural barriers and degradation of wintering sites in causing higher mortality rates as recorded for a variety of long-distance migrants. Our work confirms variation in demographic parameters across winter distribution ranges and reinforces the importance of longitudinal studies to better understand the complex demographics of migratory species.

**Supplementary Information:**

The online version contains supplementary material available at 10.1007/s00442-024-05518-x.

## Introduction

Seasonal migration is a fascinating phenomenon in which animals travel between sites to take advantage of seasonal peaks in resources (Dingle 1980; Alerstam and Lindström 1990; Newton 2008; Lok et al. 2015). However, migration is a challenging process (Alerstam et al. 2003), and different migratory decisions (e.g., migratory route and the selection of wintering site) may be associated with different fitness outcomes (e.g., Alves et al. 2013).

The considerable variation within and between species in wintering site use (Alerstam et al. 2003; Newton 2008) has been suggested to affect demographic parameters, including survival and productivity (via carry-over effects) (Alves et al. 2013; Lok et al. 2015; Grist et al. 2017; Reid et al. 2020; Acker et al. 2021; Carneiro et al. 2021). Such associations could be due to a mixture of geographically and ecologically distinct environments, experienced by individuals at their wintering site or during migration (Hötker 2003; Boyle 2008; Gillis et al. 2008; Chapman et al. 2011; Harrison et al. 2011; Sergio et al. 2014; Loonstra et al. 2019; Swift et al. 2020). For instance, individuals of Pied avocets (*Recurvirostra avosetta*) wintering closer to the breeding site arrived earlier and fledged more chicks than those wintering further away (Hötker 2003). Conversely, in Icelandic Black-tailed godwits (*Limosa limosa islandica*), those wintering furthest arrived earlier at the breeding area (Alves et al. 2012), suggesting that distance by itself cannot explain variation on arrival dates, which in seasonal environments tends to be positively related to productivity (Alves et al. 2019; Morrison et al. 2019). Partial migration is an extreme case of within species variation (Newton 2008; Chapman et al. 2011), where some individuals do not migrate at all (residents), while others do, which potentially leads to variation in key fitness components like survival (Gillis et al. 2008; Kokko 2011). For example, resident American dippers (*Cinclus mexicanus*) were recorded to have higher annual productivity than migratory individuals, and slightly lower survival, indicating a possible trade-off between productivity and survival (Gillis et al. 2008).

Nevertheless, the underlying mechanisms linking survival and wintering site remain poorly understood, owing to the difficulty in following individual birds throughout their annual cycles (

Lok et al. 2015). In fact, despite some studies showing the benefits of a shorter migration, in other systems, migrating further can be advantageous in terms of survival (Alves et al. 2013; Reneerkens et al. 2020) and/or productivity through carry-over effects (Lourenço et al. 2008; Carneiro et al. 2021). Additionally, the costs and benefits of wintering location can also vary depending on the age of the individual and the environmental conditions experienced at the wintering sites. This is the case for the Greater flamingo (*Phoenicopterus roseu*s), where long-distance migration appears to be costly for young and inexperienced individuals, but beneficial for adults (Sanz-Aguilar et al. 2012). Furthermore, the survival of resident flamingos in Southern France seems to be severely impacted by cold spells estimated to occur every 25 years (Sanz-Aguilar et al. 2012).

The Eurasian spoonbill (*Platalea leucorodia leucorodia*, hereafter spoonbill) is a migratory waterbird species, distributed from the East-Atlantic Coast to the Southeast Asia (Triplet et al. 2008). This species has been extensively monitored due to being easily detected (Triplet et al. 2008; Pigniczki and Végvári 2015) and its potential as an umbrella species (Jin et al. 2008; Schneider-Jacoby 2008; Lorenz et al. 2009) for ecosystem-level conservation of wetland areas (Sergio et al. 2006, 2008).

In Europe, breeding spoonbills are currently divided in two distinct meta-populations (Lok 2020), each using different wintering sites located along different flyways: the East Atlantic Flyway meta-population (hereafter EAF population), which is steadily increasing at several breeding sites; and the Central European Flyway meta-population, which is undergoing a moderate decline (hereafter CEF population; (Champagnon et al. 2019b)). During the past decade, the population dynamics and wintering site use of the Dutch breeding population have been intensively studied, thereby providing detailed insight into the demography of the EAF population (Lok et al. 2011, 2013a, 2017). In this population, long-distance migrants had lower survival and reproductive output compared to short-distance migrants (Lok et al. 2011, 2017). In contrast, much less is known regarding the CEF population, despite several efforts to better understand the migration and dispersal patterns of individuals from several breeding colonies in Croatia, Hungary, and Italy (Azafzaf et al. 2006; Mikuska et al. 2006; Kralj et al. 2012; Pigniczki et al. 2016, 2020; Pigniczki 2022). In this flyway, apparent survival was estimated for an expanding breeding colony in Italy (Tenan et al. 2017), however, no comparison between survival rates of individuals with different wintering sites was investigated.

The EAF and CEF populations were initially assumed to breed allopathically (i.e. not to overlap in the breeding area) (Brouwer 1964; Müller 1984), but by the end of the twentieth century, breeding adults from both populations (EAF from Netherlands and CEF from Italy) settled in Camargue, Southern France (Blanchon et al. 2010). Individuals from this recent colony currently migrate along two distinct flyways: within the EAF, following a south-western route to Spain and West Africa; or within the CEF, following a south-eastern route to Italy and Tunisia. Additionally, some individuals from this colony remain in Camargue all year around (i.e. residents) (Blanchon et al. 2019). Due to their wide array of migratory routes and distances, including residency, spoonbills from Camargue present a unique opportunity to not only further investigate the relationship between survival rates and migratory distance across wintering sites, but also to compare survival rates within and between different flyways, shedding light on the potential effects of crossing ecological barriers on survival, such as the Mediterranean Sea and the Sahara Desert.

Here, using resighting data collected at the main breeding area in Camargue and throughout the wintering range, we applied capture-mark-recapture models (Lebreton et al. 1992; Pradel 2005) to explore the relationships between survival rate, migratory flyway and distance. (1) We compared the survival rates of individuals using different flyways (resident, EAF migrant and CEF migrant), predicting that, as suggested by the contrasting populations trends (Champagnon et al. 2019b), CEF migrants have lower survival rates than EAF migrants, potentially associated with habitat degradation in wintering sites such as Tunisia (Observatoire Tunisien de l’Environnement et du Développement Durable (OTEDD), 2016). (2) We also explored the relationship between survival and migration distance, distinguishing long-distance vs short-distance migrants vs resident individuals, predicting that survival declines with increasing migration distance. In both flyways, only long-distance migrants encounter natural barriers (Sahara Desert—EAF/Mediterranean Sea—CEF), whose crossing involves high energy demands and few (or no) possibilities for emergency stopovers if needed, possibly leading to lower survival compared to short-distance migrants or resident individuals. The potential impact of crossing such barriers was also suggested by the lower survival rates of the long-distance migrants from Dutch colonies, likely influenced by the Sahara crossing during pre-breeding migration (Lok et al. 2015). (3) Finally, we assessed the interactive effect of flyway and distance by comparing differences in survival between birds with different wintering strategies (i.e., short-distance EAF migrants, long-distance EAF migrants, short-distance CEF migrants and long-distance CEF migrants, residents).

## Material and methods

### Study population and data collection

This study is based on a long-term colour-ringing program on the breeding population of spoonbills in the Camargue, which settled in 1998 and reached more than 300 breeding pairs in 2018 (Blanchon et al. 2019; Marion 2019). The Camargue is a semi-natural region of 150,000 ha making it the largest wetland in France (Roche et al. 2009). Here, spoonbills breed mainly on two small islands in a protected coastal lagoon (*Étang des Impériaux*—N43°28, E4°28).

Each year (from 2008 to 2020), between April and July, approximately 200–300 chicks around 20–25 days of age (prior to fledging) were fitted with a metal ring (with a unique alphanumeric code; FRP scheme) on one leg and an engraved PVC ring (white ring with a unique set of four black characters) on the other leg, to allow individual visual identification from a distance using a telescope or a camera (i.e., resighting). To minimize the risk of chick mortality, ringing operations occurred in the early morning (avoiding heat stress) and only under favourable weather conditions (i.e., no precipitation and/or strong winds).

From 2008 onwards, observations of previously marked individuals were performed during the incubation and early chick-rearing phase using a telescope at the breeding colony. Since 2016, these efforts were complemented with automated camera traps placed in the colony and moved regularly to survey different nests in different sections of the colony. For winter resightings, we relied on a large network of amateur and professional ornithologists and nature photographers, supplemented by dedicated expeditions to major wintering sites where resighting effort was low (i.e., Banc d’Arguin and Tunisia).

### Data selection

A total of 3540 chicks were considered for this analysis (Online Resource 1: Table S1 for further details), which resulted in ca. 16,000 resightings, ca. 10,000 of which during the breeding season (March to July) and ca. 3,000 during the winter (October to February). We excluded from our analysis: (1) individuals ringed as adults (*N* = 2), due to their low number; (2) recoveries (*N* = 155) that mainly encompassed chicks that died before fledging and ring recoveries of which we did not know the time since death or if it corresponds to ring loss; (3) individuals fitted with GPS tags, as these could have a potential effect in survival (*N* = 21) (Pennycuick et al. 2012; Weiser et al. 2016; Bodey et al. 2018); and (4) duplicated rings that originated from a fieldwork error in one year (*N* = 22). During the breeding season only resightings from the *Étang des Impériaux* were considered and limited to one observation per individual per year, considering only records with no reading uncertainty, thus resulting in *N*_obs_ = 1527 resightings.

**Fig. 1:**
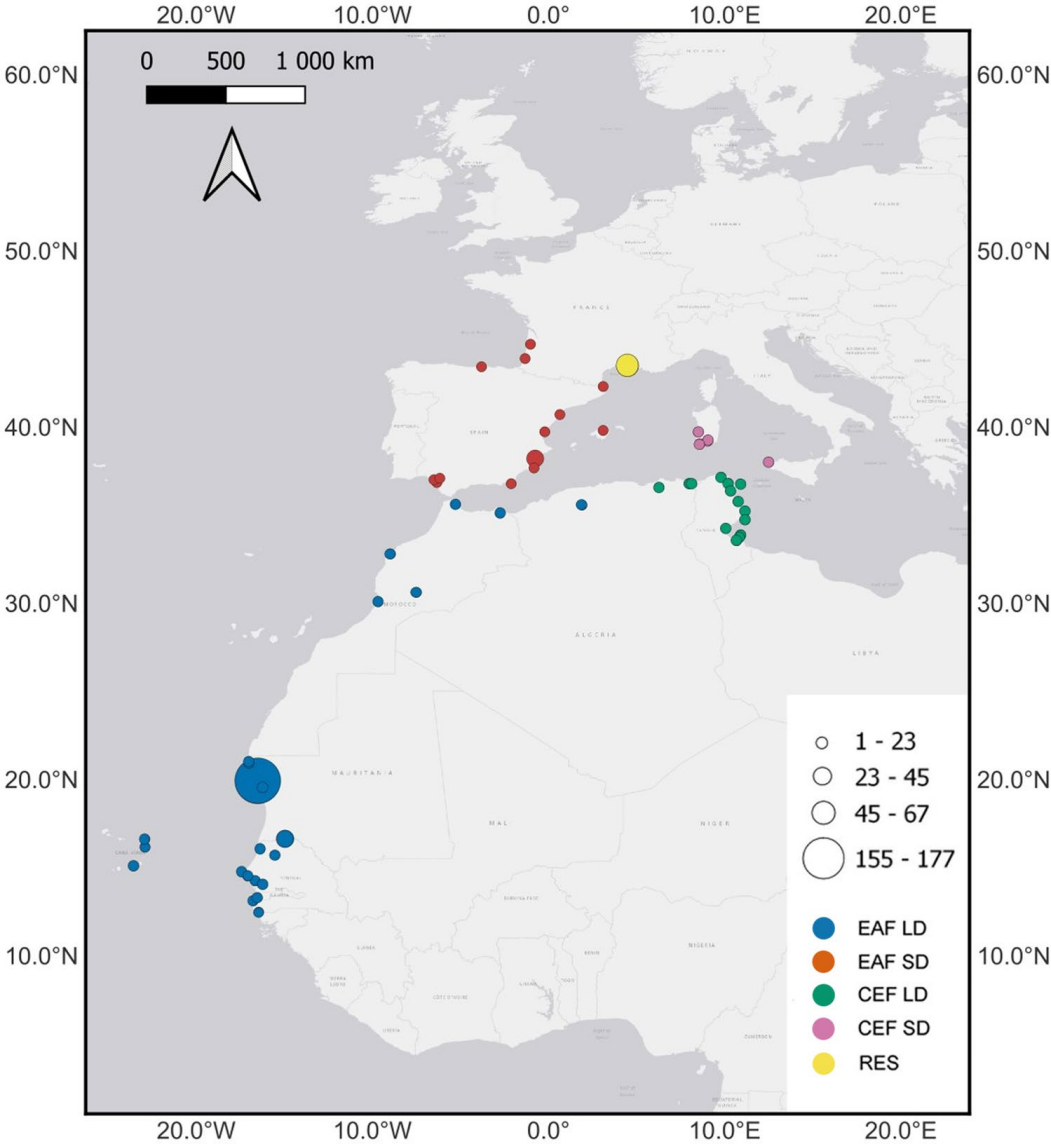
Distribution of the 484 individuals (size of circle indicates numbers of individuals) according to their southernmost wintering site and flyway: EAF LD—long-distance migrants East-Atlantic Flyway: Cape Verde, Gambia, Morocco, Mauritania, Senegal, and West Algeria; EAF SD—short-distance migrants East-Atlantic Flyway: Portugal, Spain and Southwest France; CEF LD—long-distance migrants Central European Flyway: East Algeria and Tunisia; CEF SD—short-distance migrants Central European Flyway: Italy; RES—residents in Camargue, France. Note: two individuals resighted in West Algeria were considered as EAF LD and not CEF LD, as recent GPS data of tagged individuals shows that some of the birds migrate through West Algeria while following the EAF (Lok 2021)

We defined five wintering regions, considering both the migratory flyway (East Atlantic Flyway—EAF; Central European Flyway—CEF) and the distance travelled (long-distance—LD; short-distance—SD; and residents—RES; Fig. 1 and Online Resource 1: Table S1).

Individuals were assigned to a wintering region according to the site where they were resighted during winter, which was defined as the period between October and February for long-distance migrants. To avoid the possible misclassification for the short-distance migrants (and resident) individuals due to a late-autumn or early-spring stopover resighting, we only considered winter resightings during the months of November to January (Navedo et al. 2010; Lok et al. 2011). In our analysis, we assumed that individuals do not change wintering site (Lok et al. 2013a). This was done to avoid overparameterization of the models with state uncertainty due to the small number of such records (*N* = 24) (Pradel 2005). If an individual was observed at different sites within the same flyway, either within the same winter or in different winters, we selected the southernmost site as its winter site (*N* = 10). We excluded birds that changed their migratory flyway or switched from being migratory to resident or vice versa (*N* = 14). Three individuals resighted in South Sudan were excluded as these did not match any of the main wintering areas and their number was very limited. Since the main objective of this study was to understand how wintering strategies affect the survival of the Camargue spoonbills, we only considered individuals for which their wintering sites were known. Therefore, we excluded birds that were not seen in winter. Hence, the first winter resighting marked the start of an individual’s encounter history, thus resulting in 484 individuals distributed among the various wintering strategies (Online Resource 1:Table S1).

### Mark-recapture modelling & statistical analysis

To estimate the survival rates of spoonbills, we developed Cormack–Jolly–Seber models (CJS) (Cormack 1964; Jolly 1965; Seber 1965) in E-surge v. 2.2.3 (Choquet et al. 2009b), which provided a flexible modelling framework to develop, constrain and rank complex mark-recapture models (Pradel 2005, 2009).

In our study, all individuals have an initial state of alive at the wintering site, hence the initial state probability was fixed to “1”. As for the transition probabilities, we assumed that our individuals did not change wintering and breeding sites, and therefore the only transition probability considered was in fact survival probability (Lok et al. 2013a, b). As we only used resightings in the Camargue to estimate survival, permanent emigration from this breeding site cannot be distinguished from mortality (Lebreton et al. 1992). As such, the resulting estimates reflected “apparent” or “local” survival (Lebreton et al. 1992). Finally, the models also estimated the probability of being resighted at the *Étang des Impériaux* (*p*) during the breeding season. Since the initial state is alive at the wintering site and only resightings at the breeding site were considered in subsequent occasions, survival during the first interval (hereafter *Φ*^1^) corresponds to the probability that an individual survived from the winter when it was first observed until the next breeding period (for details regarding the age at first sighting, see Online Resource 1: Table S2). Therefore, the first interval is shorter (half a year) than the subsequent intervals that correspond to a full year, from one breeding season to the next (hereafter *Φ*^2+^). Due to lack of continuous data, subsequent resightings obtained at wintering sites were not used. As a result, *Φ*^2+^ reflects the apparent survival of birds that survived and returned to the breeding grounds after their first winter observation, while *Φ*^1^ includes any residual mortality occurring prior to return to the breeding grounds, or permanent emigration from the breeding grounds, that cannot be explained by the *Φ*^2+^ estimates.

In capture-mark-recapture models, the goodness-of-fit test allows to assess if the data does not infringe any assumption of parameter homogeneity (Burnham et al. 1987; Lebreton et al. 1992; Pradel et al. 1997). We used program U-Care V2.3.4 (Choquet et al. 2005, 2009a) to test the goodness-of-fit of the CJS model *Φ*_g*t_* p*_g*t_ to the data, with the five previously defined wintering regions (Fig. 1) as groups (*g*). This test model does not account for time-since-marking (or in this case, time since the first winter sighting) or age effects. TEST3sr estimated a lack of fit possibly caused by transients and/or the shorter first time interval (6 months) compared to later intervals (12 months; *χ*^*2*^ = 126.07*, df* = 26*, P* ≤ 0.001) which was accounted for by separately estimating *Φ*^*1*^ and *Φ*^*2*+^(Pradel et al. 1997). The remaining lack of fit (as estimated by TEST2 and TEST3sm) was accounted for by adjusting for overdispersion (*ĉ* = *χ*^*2*^*/df* = 93.56/68 = 1.38) (Choquet et al. 2005).

As reported by Lok et al. (2017, 2009) in previous studies and substantiated by observations at the study colony (Champagnon et al. 2019a), spoonbills have delayed maturity and usually start breeding in their fourth calendar year (*cy*) (Cramp and Simmons 1977). Until reaching maturity, spoonbills usually stay at the wintering grounds (but see below). Combined with the fact that the first interval after the first winter sighting is only half a year, the goodness-of-fit model (Model 1, Online Resource 1: Table S3) does not correspond to our general biologically meaningful model. In our general model, survival was constrained as a function of the following explanatory variables: (1) time since first winter observation (categorical with two levels: *Φ*^*1*^, half year interval from first winter observation to next breeding season vs* Φ*^*2*+^*,* subsequent one-year intervals); (2) age class (*2age*, categorical with two levels: immatures (0.5–3.5 cy) and adults (4 + cy)). Individuals were categorized in groups according to their age at first winter resighting, which allowed us to estimated age-specific survival and resighting probabilities. For example, if an individual was observed for the first time in winter in its 1st winter, it starts its encounter history as 0.5 cy, but will be considered an adult (4 + cy) as soon as it reaches its fourth breeding season; (3) wintering region (a categorical interaction of migratory flyway and distance (dis*fly), resulting in five levels: EAF LD, EAF SD, CEF LD, CEF SD and RES); and (4) year (*t,* categorical with 12 levels). Resighting probability was modelled as a function of: (1) three age classes (*3age*, categorical with three levels: 2 cy, 3 cy and 4 + cy (adult)). Although spoonbills usually remain at the wintering grounds through their third winter (until reaching maturity), some immature birds do return to the colony and can thus be resighted (Boulinier et al. 1996, 2008; Johnstone et al. 2002; Lok et al. 2013b; Tenan et al. 2017); (2) annual variation (*t*, categorical with 13 levels); and (3) migration distance (*dis:* LD; SD; RES), as long-distance migrants may have a shorter stay at the breeding sites, owing to delayed arrival in spring and/or earlier departure in autumn. This could translate into a shorter probability of being resighted compared to short-distance migrating or resident birds (Lok et al. 2013a). Although annual variation was included, between 2008 and 2013, the estimates were fixed to zero due to the absolute lack of resightings despite field efforts. As an alternative to modelling annual variation in *p*, we considered models in which *p* differed between three periods according to whether camera traps were used (*ct*): (1) 2008–2013 fixed to zero; (2) 2014–2015, efforts without camera traps; and (3) 2016–2020, efforts with camera traps. Our final full complex model is *Φ*^*1*^_2age*dis*fly*t_* Φ*^*2*+^_2age*dis*fly*t_* p*_3age*dis*fly*t_. However, only the interactions between *dis* and *fly* (EAF; CEF; RES) were considered afterwards, as testing interactions with year and age led to problems of parameter identifiability due to the data being scarce for some wintering strategies, years, and age classes. Based on previous findings (Lok et al. 2013b), we accounted for a potential effect of age on survival and resighting probabilities in all models. Subsequently, different models were developed and variables of interest compared following a *stepwise* approach with a two-step process (Lebreton et al. 1992; Anderson and Burnham 2002; Grosbois and Tavecchia 2003). First, we kept survival fully parameterized while we constrained the parameterization of resighting probability (*p*). Using the best-supported parameterization of *p,* we then constrained the parameterization of survival probabilities (Grosbois and Tavecchia 2003; Doherty et al. 2012) during the first and**/**or subsequent intervals (*Φ*^*1*^ versus* Φ*^*2*+^). To confirm the robustness of our selection procedure, as the survival and resighting probability parameters are not entirely independent in these models, we repeated this process, while reversing the order of the two steps by first constraining the parameterization of survival probability and then of resighting probability (Doherty et al. 2012). Parameter estimates and profile likelihood confidence intervals from the best-supported model are reported. Model selection was based on the Akaike Information Criterion, adjusted for small sample sizes and overdispersion (QAIC_c_), considering a better model fit between competing models when *ΔQAIC*_*c*_ < 2 (Anderson and Burnham 2002). When there were multiple models within two QAIC_**c**_ points of the best-supported model, the model with the fewest parameters was selected (i.e., the most parsimonious model, Anderson and Burnham 2002).

### Permanent emigration cases

To approximate true survival, we calculated the possible cases of permanent emigration in our dataset. For individuals that were not resighted again after their first winter sighting, we checked whether they were subsequently seen during spring or summer (March to July) en route to/from or at breeding sites outside the Camargue in the EAF and the CEF. If so, we considered them as a ‘possible permanent emigration case’. From these cases, we calculated the ‘minimal’ permanent emigration probabilities per wintering region and divided it by the total number of individuals never seen again per wintering region. To approximate our results to true survival, we used these estimates to correct the estimated apparent survival per wintering region (=( *Φ*^*1*^ wintering region)/(1−% permanent immigration of wintering region)).

## Results

**Fig. 2:**
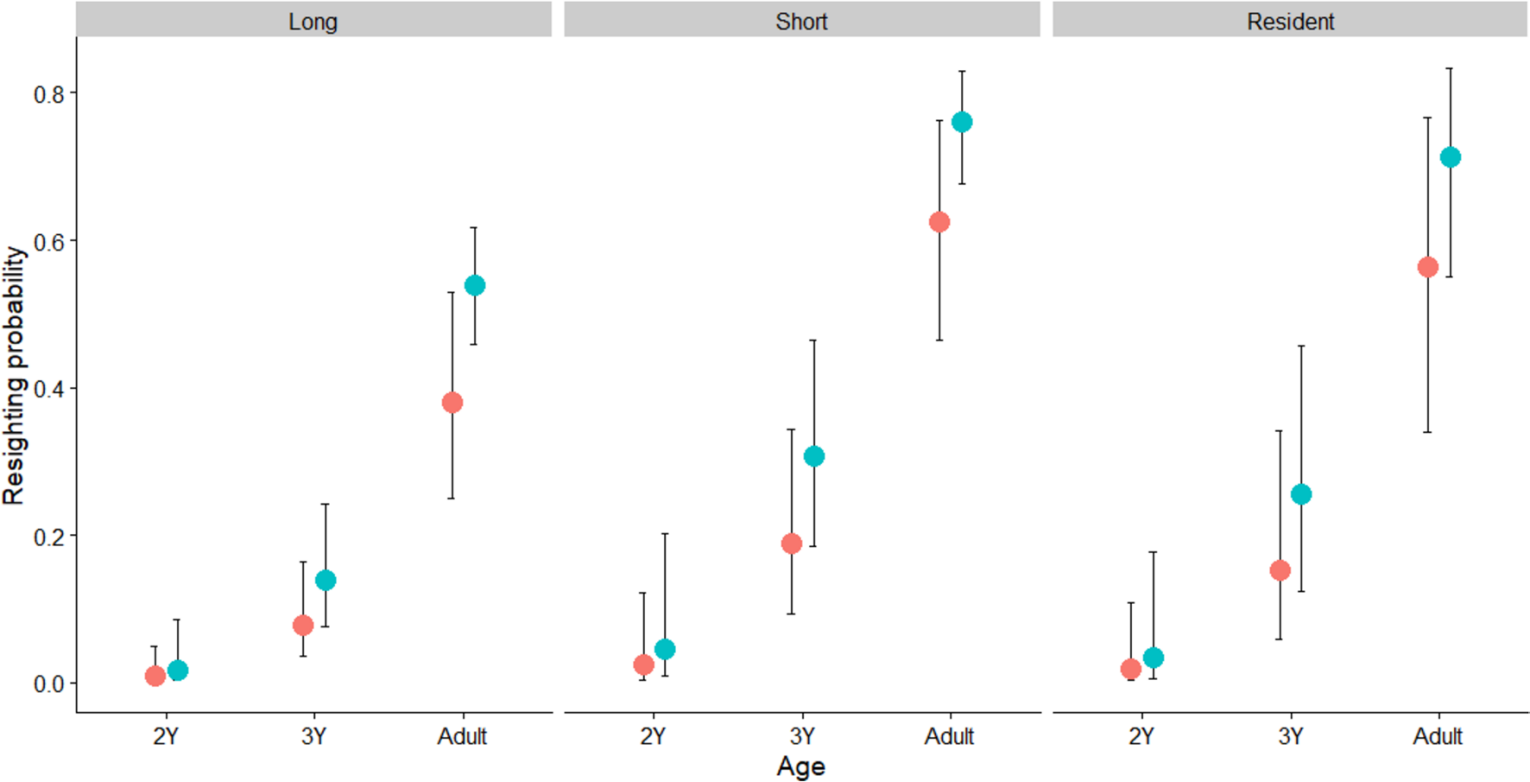
Resighting probability of spoonbills according to age class (*2Y* = 2 calendar-years; *3Y* = 3 calendar-years; AD = adult), use of camera traps (red—no camera trap used; green—camera trap used) and migration distance (Long; Short; Resident). Estimates are based on the best-supported model from Table 1 (Model 100). Vertical lines indicate 95% confidence intervals

When applying the *stepwise* approach by first constraining the resighting probability, the best-supported parameterization for resighting probability included camera trap use (*ct*), three age classes (*3age*) and migration distance (*dis*) effects (Model 4, Online Resource 1: Table S3). Resighting probability was higher in years when camera traps were used, lower for long-distance migrants and increased with age (Fig. 2).

Using the best-supported parameterization of resighting probability, three competitive parameterizations for survival with QAIC_c_ < 2 were identified, which included either an effect of migratory flyway and/or distance on survival during the first half-year interval (*Φ*^*1*^, Table 1). The best-supported and most parsimonious model contained an effect of age and migration distance on *Φ*^*1*^ and of age on *Φ*^*2*+^, with *ΔQAIC*_*c*_ = –13 compared to the model without an effect of migration distance (Model 110, Online Resource 1: Table S3). The same most parsimonious model was selected when applying the reverse *stepwise* approach (Model 104, Online Resource 1: Table S4).

**Table 1:** Model results of survival probability from the stepwise approach

Model	*ϕ*^1^	*ϕ*^2+^	*K*	Deviance	ΔQAIC_c_	Akaike weight
***100***	***2age* + *dis***	***2age***	**12**	**1397.9**	0.0*	**0.37**
*70*	*2age* + dis*fly	*2age*	14	1392.7	0.3	0.34
*80*	*2age* + *dis* + *fly*	*2age*	13	1397.3	1.7	0.19
*98*	*2age* + *dis*	*2age* + *fly*	14	1396.8	3.3	0.04
*99*	*2age* + *dis*	*2age* + *dis*	14	1397.4	3.8	0.02
*68*	*2age* + dis*fly	*2age* + *fly*	16	1392.2	4.1	0.01
*69*	*2age* + dis*fly	*2age* + *dis*	16	1392.2	4.1	0.01

*Φ*^*1*^ apparent survival first half year, *Φ*^*2*+^ apparent survival subsequent full years,* K* number of parameters, *2age* 2 age classes: immatures (0.5–3.5 cy) and adults (4 + cy), *dis* migration distance, dis*fly wintering region, *fly* migratory flyway

*QAIC_c_ = 1040.35. Models were ranked according to the Akaike weight and only models with an Akaike weight of ≥ 0.01 are shown (the complete model results are shown in Online Resource 1: Table S3). The best-supported model is indicated in bold

**Fig. 3:**
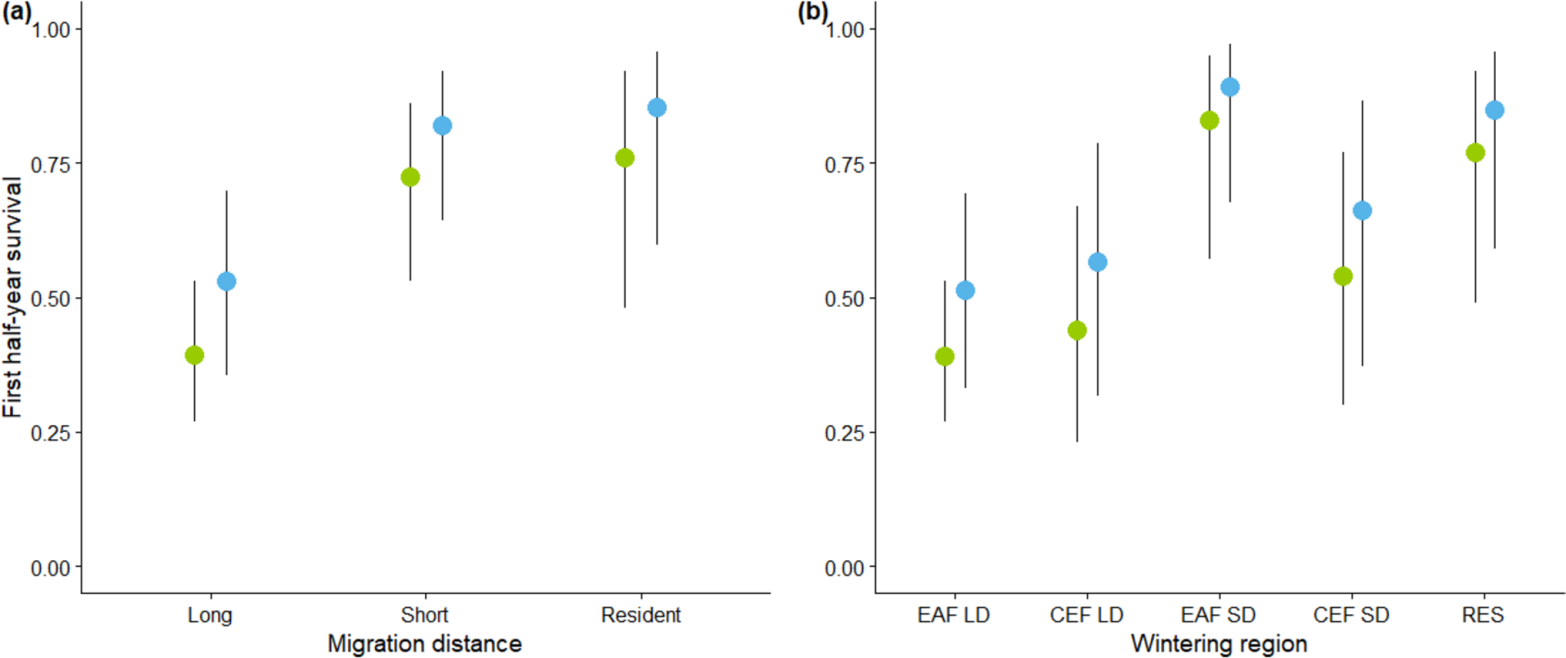
Apparent survival estimates of immature (green) and adult (blue) spoonbills during the first half-year interval (*Φ*^*1*^): (**a**) according to migration distance (long; short; resident) as estimated by the best supported model of the Table 1 (model 100, *Φ*^*1*^: *2age* + *dis*); and (**b**) according to the wintering region (East Atlantic Flyway long-distance—EAF LD; Central European Flyway long-distance—CEF LD; East Atlantic Flyway short-distance—EAF SD; Central European Flyway short-distance—CEF SD; resident—RES) as estimated by the second-best model in Table 1 (Model 70, *Φ*^*1*^: *2age* + dis*fly). The *2age* variable corresponds to two age classes: immatures (0.5–3.5 cy) and adults (4 + cy). Vertical lines indicate 95% confidence intervals

Apparent survival of spoonbills during the first half-year interval (*Φ*^*1*^) was lower for long-distance migrants compared to residents and short-distance migrants, who had similar survival (Fig. 3a). When considering the second-best model (Table 1, Model 70), which included the interaction between migration distance and flyway, survival of short-distance migrants appeared to differ between flyways, with CEF SD having lower survival than EAF SD (Fig. 3b). There was no support for an effect of migratory flyway or distance on survival after the first half year period (*Φ*^*2*+^).

When considering the possible permanent emigration cases of Table 2, permanent emigration was estimated to be highest in EAF LD (11%), intermediate for EAF SD and CEF SD (7% and 6%) and very low for CEF LD (1%) and RES (2%). This would imply a “true survival” rate of 0.37 and 0.58 for immatures and adults performing long-distance migration, which is still considerably lower than the estimates of 0.77 and 0.88 for immature and adult short-distance migrants and 0.79 and 0.87 for resident birds.

**Table 2:** Number of individual spoonbills wintering in each region, number of individuals never recorded again after their first winter resighting at the breeding site in Camargue and number of individuals observed on their way from/to or at breeding sites other than Camargue

Wintering region	Total	Not recorded again	Possible permanent emigration cases	
			Number of individuals	%^a^
EAF LD	257	195	29	11%
EAF SD	71	26	5	7%
CEF LD	75	57	1	1%
CEF SD	33	19	2	6%
RES	48	19	1	2%

*EAF LD* East Atlantic flyway long-distance, EAF SD East Atlantic Flyway short-distance, *CEF LD* Central European Flyway long-distance, *CEF SD* Central European Flyway short-distance, *RES* resident

^a^Percentage was calculated by dividing the number of potentially permanently emigrated individuals by the total number of individuals in each winter region

## Discussion

Our results do not support differences in survival rates of Camargue spoonbills following different migratory flyways, as flyway was not included in the best-supported model. This contradicts our initial prediction that individuals using the Central European Flyway would have lower survival than the individuals with other migratory routes or behaviours, given the apparent decreasing trend of this population and the degradation of wintering sites in Tunisia (Observatoire Tunisien de l’Environnement et du Développement Durable (OTEDD), 2016; Champagnon et al. 2019b). Yet, confirming our initial prediction, we did find a correlation between migration distance and apparent survival, but only in the first half year, and not in subsequent years.

As we only use resightings performed in Camargue, our estimates of *Φ*^*1*^ and *Φ*^*2*+^ reflect apparent survival, which is the product of true survival and permanent emigration. Although highly philopatric, breeding dispersal of spoonbill is not excluded. Permanent emigration from the Camargue to other European breeding sites may occur (Lebreton et al. 1992; Cilimburg et al. 2002; Lindberg et al. 2007). Like White storks (*Ciconia ciconia*) (Chernetsov et al. 2004), spoonbills are social birds, also during their migration (de Goeij et al. 2012; Navedo and Garaita 2012; Lok et al. 2019), where social interactions likely influence the decisions of immatures regarding when and where to migrate (Aikens et al. 2022). When wintering along the Atlantic coast of Africa (EAF LD region—Cape Verde, Gambia, Mauritania, Morocco, Senegal, and West Algeria), individuals from Camargue mix with spoonbills from other EAF breeding populations (e.g., Dutch & Iberian). Likewise, individuals wintering in Northeast Africa (CEF LD region—East Algeria and Tunisia) mix with spoonbills from breeding populations like Hungary and Italy. Thus, immatures from Camargue could possibly follow adults from other breeding populations to their breeding sites in South- and North-Western Europe (EAF) or Italy and Central Europe (CEF).

The lower apparent survival in *Φ*^*1*^ of long-distance migrants may be partially driven by a higher probability of permanent emigration of Camargue spoonbills to other breeding sites (Lebreton et al. 1992; Cilimburg et al. 2002; Lindberg et al. 2007). Nevertheless, despite considerable observation efforts along the migratory flyways and in other European breeding sites (Lok et al. 2013a; Pigniczki 2017), there are few records of Camargue spoonbills (*N* = 38) resighted along migratory routes to, or in, other breeding sites (Table 2). If all those 38 cases correspond to spoonbills that permanently emigrated, EAF LD migrants had in fact a 4–10% higher probability of permanent emigration compared to other migration strategies. Nevertheless, while this can partly explain the lower apparent survival of EAF LD migrants, it does not fully explain the differences in survival between long-distance and short-distance migrants.

To estimate *Φ*^*2*+^, only resightings in Camargue during the breeding season were considered. Thus, *Φ*^*2*+^ reflects survival of individuals that survived and returned to the breeding site at least once after their first winter observation. Most birds were still relatively young (≤ 5 years old, Online Resource 1: Table S2) when seen for the first time at their wintering sites. As spoonbills usually only start breeding when they are 4 years old, and usually stay at the wintering site until that age, most of these individuals are yet to make their first return migration. Therefore, our finding that the effect of migration distance was only supported for *Φ*^*1*^ and not *Φ*^*2*+^ could imply that the increased mortality of long-distance migrants primarily occurred before their first return to the breeding grounds, i.e., either at the wintering grounds or during the first return migration. Mortality may also have been higher for juveniles attempting to migrate long distances during their first southward migration compared to resident or short-distance migrating juveniles, but since our analysis started at the first winter sighting, this potential effect on juvenile survival could not be estimated. After their first return migration, spoonbills appeared experienced enough to be equally likely to survive the next annual cycle independently of where they winter. In fact, the apparent survival of adult Camargue long-distance migrants after the first half-year [${\Phi }_{ad}^{2+}$= 0.94, 95%CI (0.88–0.96)] is higher than that of long-distance migrants in the Dutch population (Lok et al. 2015). This partly supports our hypothesis that despite the lower apparent survival of long-distance migrants being mainly driven by higher mortality, with experience, spoonbills seem to successfully respond to migration challenges independently of the wintering region used, although direct comparisons with other studies should be performed with care.

As shown in other studies (Alves et al. 2013; Lok et al. 2015), the variation in survival detected in our study is not fully explained by migration distance. In fact, there are likely additional factors contributing to the observed variation in mortality, such as the crossing of natural barriers and habitat degradation. Reaching the wintering sites south of the Sahara clearly involves travelling a longer distance than reaching Tunisia, yet no difference in survival between these two wintering regions was detected in the top-ranking models. To explore the possible role of crossing natural barriers in survival, namely the Sahara Desert in the East Atlantic Flyway and the Mediterranean Sea (between France, Sardinia, and Tunisia) in the Central European Flyway, we plotted the survival estimates of the second-best model which contained the interaction between flyway and distance in *Φ*^*1*^ (Model 70 in Table 1; Fig. 3b). Individuals wintering in the CEF SD region are mainly wintering on the island of Sardinia and had slightly lower survival than the ones in the EAF SD, which are mainly wintering in Spain. To our knowledge, the EAF SD migrants that migrate to South-Western France or Spain do not cross any major natural barrier, thus the lower survival of the CEF SD suggests the possible impact of partially crossing the Mediterranean Sea to Sardinia. Furthermore, the high and similar survival estimates of individuals wintering in the EAF SD region and those resident to the Camargue, could be due to the lack of natural barriers in both strategies.

Habitat degradation has also been reported to cause population declines in migratory species (Morrison et al. 2001; Piersma et al. 2016; Studds et al. 2017). Hence, the deterioration of wintering and stop-over sites in Africa could amplify the difficulties of undertaking a long-distance migration (Brochet et al. 2016) that involves the crossing of natural barriers. In contrast, in some European wintering sites, changes in water management as well as enforced wetland protection (e.g., European Union’s Birds and Habitats Directives; Water Framework Directive—2000/60/EC) are likely to have improved stopover and wintering conditions in Europe (Novo and Cabrera 2006; Donald et al. 2007). Additionally, winter temperatures in France and Iberia have increased over the last decades, which reduced the thermoregulation costs of spoonbills wintering in Europe and may also have increased food availability (Klein et al. 2002; Lok et al. 2013a; Shukla et al. 2019).

Although the migratory flyway effect was not included in the best-supported model, the second-best model included an interaction between migration distance and flyway, implying that the distance effect on survival differed depending on the flyway taken. This model (Model 70, Table 1) estimated similar survival for EAF LD, CEF LD and CEF SD migrants that was lower than the survival of EAF SD and RES birds (Fig. 3b). That this was not the best-supported model may be due to the relatively low sample sizes of birds with known wintering site in the Central European Flyway (Online Resource 1: Table S1), causing large confidence intervals around the survival estimates of CEF migrants (Fig. 3b). Assuming that spoonbills from other CEF breeding sites experience similarly low survival as we estimated for the CEF SD and CEF LD migrants from the Camargue, while a high and increasing proportion of the EAF spoonbills use high survival regions in France and Spain (Lok et al. 2013a), this could explain the contrasting population trends of the CEF (slightly declining) versus the EAF (increasing) meta-population (Champagnon et al. 2019b). The decline of the CEF meta-population is mainly driven by the Hungarian population (Champagnon et al. 2019b), from which spoonbills are mainly seen wintering in Gulf of Gabes (Tunisia, Pigniczki 2022). Nevertheless, spoonbills from the Italian population have high adult survival rates [0.91, 95%CI (0.85–0.96)] and this population is currently increasing (Tenan et al. 2017), contrasting to the overall slight decline of the entire CEF meta-population. To better understand the causes of the different population trends, we strongly advocate for an analysis combining the several interconnected spoonbill populations throughout Europe, not only to get more precise survival estimates for the different wintering regions, but also to estimate the proportion of birds from the CEF and EAF populations wintering in regions associated with relatively low or high survival.

Despite considerable variation within and among species in wintering site use being previously shown to affect demographic parameters, few longitudinal studies have been able to compare the survival rates of a population using different flyways and migratory ranges (specifically contrasting residency vs short-distance vs long-distance). While confirming previous studies by Lok and collaborators that indicated lower survival of long-distance migrating spoonbills (Lok et al. 2011, 2013a, 2015), this study is the first to indicate that such survival cost becomes apparent during the first return migration. Additionally, our results show that spoonbills seem to cope better with migratory challenges and wintering conditions as they age. Our study therefore highlights the heterogeneity in demographic parameters across wintering ranges, but also the relevance of long-term studies to better understand the complex demography of a migratory species and thus help prioritise conservation actions according to population dynamics and connectivity.

## Author contribution statement

HF, JA, JC, TL conceived and designed the study. HF carried out the analysis and wrote the first draft, followed by substantial contributions from JA, JC, and TL. HF, JA, JC, and TL critically revised the analysis and manuscript, and all authors approved its submission.

### Supplementary Information

Below is the link to the electronic supplementary material.

Supplementary file1 (PDF 415 KB)

## Data Availability

The datasets generated during and/or analysed during the current study are available in the Zenedo repository, https://doi.org/10.5281/zenodo.7576378.
