# Supplementary material for: Relationship between wintering site and survival in a migratory waterbird using different migration routes
Source: Oecologia. 2024 Feb 24;204(3):613–24. doi: 10.1007/s00442-024-05518-x (PMC10980637; doi:10.1007/s00442-024-05518-x)
Supplement: Supplementary file 1 — Supplementary file1 (PDF 415 KB) [file 442_2024_5518_MOESM1_ESM.pdf]

Electronic Supplemental Material (ESM)

Journal name: Oecologia
Manuscript Title: *Relationship between wintering site and survival in a migratory waterbird using different migration routes*
Authors: Hugo R. S. Ferreira, Jocelyn Champagnon, José A. Alves, and Tamar Lok

Table S1. Number of spoonbills ringed and resighted in winter, in total and per wintering region, specified per year of birth, and the number of different spoonbills resighted per wintering region during summer in *Etang des Impériaux, Camargue,* specified per year.

			Resighted per wintering region							No. of different birds resighted in *Etang des Impériaux* per wintering region				
Year of birth	Ringed	Seen in winter	EAF LD	EAF SD	CEF LD	CEF SD	RES	Summer Year	Seen in summer	EAF LD	EAF SD	CEF LD	CEF SD	RES
2008	30	4	4	0	0	0	0	2009	0	0	0	0	0	0
2009	55	24	10	11	1	2	0	2010	0	0	0	0	0	0
2010	140	35	19	12	3	1	0	2011	0	0	0	0	0	0
2011	240	57	31	10	12	1	3	2012	0	0	0	0	0	0
2012	224	67	40	6	12	3	6	2013	0	0	0	0	0	0
2013	262	101	80	14	1	4	2	2014	23	11	9	1	1	1
2014	542	114	45	12	23	14	20	2015	36	14	13	3	3	3
2015	401	133	78	18	16	7	14	2016	74	24	25	4	6	15
2016	454	53	30	8	7	3	5	2017	86	29	27	6	7	17
2017	370	44	24	5	10	3	2	2018	119	38	35	17	8	21
2018	73	32	12	4	9	4	3	2019	111	35	34	10	10	22
2019	421	54	30	5	12	4	3	2020	105	30	34	11	8	22
2020	228	24	19	0	2	0	3							

Abbreviations: East Atlantic Flyway long-distance – EAF LD; East Atlantic Flyway short-distance – EAF SD; Central European Flyway long-distance – CEF LD; Central European Flyway short-distance – CEF SD; resident – RES.

Table S2. Number of individual spoonbills seen for the first time at the wintering site by **age**.

**Age**	**Number of different individuals seen for the first time**
**1st winter (= 0.5 cy)**	227
**2nd winter (= 1.5 cy)**	90
**3rd winter (= 2.5 cy)**	57
**4th winter (= 3.5 cy)**	39
**5th winter (= 4+ cy)**	33
**>5th winter (= 4+ cy)**	38

Table S3. *Stepwise* approach, model selection results by step.

	**Model**	ϕ^1	ϕ^{2+}	***p***	***K***	**Deviance**	**$\Delta QAIC_c$**
**Step 1 - Selection on p**	**1****	*dis*fly * t*		*dis*fly * t*	87	2116.4	690.9
	**2**	*2age * dis*fly * t*	*2age * dis*fly * t*	*dis * 3age * t*	167	1197.2	241.5
	**3**	*2age * dis*fly * t*	*2age * dis*fly * t*	*dis + 3age + t*	136	1235.0	178.8
	**4**	***2age * dis*fly * t***	***2age * dis*fly * t***	***dis + 3age + ct***	**131**	**1255.4**	**179.7**
	**5**	*2age * dis*fly * t*	*2age * dis*fly * t*	*dis + 2age + ct*	130	1267.9	186.0
	**6**	*2age * dis*fly * t*	*2age * dis*fly * t*	*dis + ct*	129	1370.8	258.0
	**7**	*2age * dis*fly * t*	*2age * dis*fly * t*	*ct + 3age*	129	1276.9	189.8
	**8**	*2age * dis*fly * t*	*2age * dis*fly * t*	*ct*	127	1384.3	262.4
	**9**	*2age * dis*fly * t*	*2age * dis*fly * t*	*3age*	133	1319.3	231.7
	**10**	*2age * dis*fly * t*	*2age * dis*fly * t*	*c*	131	1474.6	338.8
**Step 2 - Selection on S**	**11**	*2age + dis*fly + t*	*2age + dis*fly + t*	*dis + 3age + ct*	37	1357.5	23.6
	**12**	*2age + dis*fly + t*	*2age + dis + fly + t*	*dis + 3age + ct*	36	1357.8	21.7
	**13**	*2age + dis*fly + t*	*2age + fly + t*	*dis + 3age + ct*	35	1358.8	20.2
	**14**	*2age + dis*fly + t*	*2age + dis + t*	*dis + 3age + ct*	35	1358.2	19.8
	**15**	*2age + dis*fly + t*	*2age + t*	*dis + 3age + ct*	33	1359.6	16.5
	**16**	*2age + dis*fly + t*	*2age + dis*fly*	*dis + 3age + ct*	29	1365.1	11.9
	**17**	*2age + dis*fly + t*	*2age + fly + dis*	*dis + 3age + ct*	28	1365.5	10.0
	**18**	*2age + dis*fly + t*	*2age + fly*	*dis + 3age + ct*	27	1366.6	8.7
	**19**	*2age + dis*fly + t*	*2age + dis*	*dis + 3age + ct*	27	1366.0	8.2
	**20**	*2age + dis*fly + t*	*2age*	*dis + 3age + ct*	25	1367.3	4.9
	**21**	*2age + dis + fly + t*	*2age + dis*fly + t*	*dis + 3age + ct*	36	1362.7	25.2
	**22**	*2age + dis + fly + t*	*2age + dis + fly + t*	*dis + 3age + ct*	35	1362.9	23.1

**23**	2age + dis + fly + t	2age + fly + t	dis + 3age + ct	34	1363.9	21.7
24	2age + dis + fly + t	2age + dis + t	dis + 3age + ct	34	1363.1	21.1
**25**	2age + dis + fly + t	2age + t	dis + 3age + ct	32	1364.5	17.8
**26**	2age + dis + fly + t	2age + dis*fly	dis + 3age + ct	28	1369.7	13.0
**27**	2age + dis + fly + t	2age + fly + dis	dis + 3age + ct	27	1369.9	11.1
**28**	2age + dis + fly + t	2age + fly	dis + 3age + ct	26	1371.1	9.8
**29**	2age + dis + fly + t	2age + dis	dis + 3age + ct	26	1370.2	9.2
**30**	2age + dis + fly + t	2age	dis + 3age + ct	24	1371.8	6.1
**31**	2age + fly + t	2age + dis*fly + t	dis + 3age + ct	35	1376.1	32.8
**32**	2age + fly + t	2age + dis + fly + t	dis + 3age + ct	34	1376.9	31.2
**33**	2age + fly + t	2age + fly + t	dis + 3age + ct	33	1386.8	36.2
**34**	2age + fly + t	2age + dis + t	dis + 3age + ct	33	1378.6	30.2
**35**	2age + fly + t	2age + t	dis + 3age + ct	31	1388.4	33.0
**36**	2age + fly + t	2age + dis*fly	dis + 3age + ct	27	1382.1	19.9
**37**	2age + fly + t	2age + fly + dis	dis + 3age + ct	26	1382.5	18.0
**38**	2age + fly + t	2age + fly	dis + 3age + ct	25	1393.9	24.2
**39**	2age + fly + t	2age + dis	dis + 3age + ct	25	1382.6	16.0
**40**	2age + fly + t	2age	dis + 3age + ct	23	1394.7	20.6
**41**	2age + dis + t	2age + dis*fly + t	dis + 3age + ct	35	1363.3	23.5
**42**	2age + dis + t	2age + dis + fly + t	dis + 3age + ct	34	1363.5	21.4
**43**	2age + dis + t	2age + fly + t	dis + 3age + ct	33	1364.4	19.9
**44**	2age + dis + t	2age + dis + t	dis + 3age + ct	33	1364.5	20.0
**45**	2age + dis + t	2age + t	dis + 3age + ct	31	1365.8	16.6
**46**	2age + dis + t	2age + dis*fly	dis + 3age + ct	27	1370.0	11.1
**47**	2age + dis + t	2age + fly + dis	dis + 3age + ct	26	1370.2	9.1
**48**	2age + dis + t	2age + fly	dis + 3age + ct	25	1371.3	7.8
**49**	2age + dis + t	2age + dis	dis + 3age + ct	25	1371.2	7.8
**50**	2age + dis + t	2age	dis + 3age + ct	23	1372.8	4.7
**51**	2age + t	2age + dis*fly + t	dis + 3age + ct	33	1383.0	33.5
**52**	2age + t	2age + dis + fly + t	dis + 3age + ct	32	1383.9	31.9
**53**	2age + t	2age + fly + t	dis + 3age + ct	31	1394.8	37.7
**54**	2age + t	2age + dis + t	dis + 3age + ct	31	1385.1	30.7
**55**	2age + t	2age + t	dis + 3age + ct	29	1397.1	35.1
**56**	2age + t	2age + dis*fly	dis + 3age + ct	25	1389.5	21.0
**57**	2age + t	2age + fly + dis	dis + 3age + ct	24	1390.0	19.3
**58**	2age + t	2age + fly	dis + 3age + ct	23	1402.9	26.6
**59**	2age + t	2age + dis	dis + 3age + ct	23	1390.4	17.5
**60**	2age + t	2age	dis + 3age + ct	21	1404.4	23.5
**61**	2age + dis*fly	2age + dis*fly + t	dis + 3age + ct	28	1378.5	19.4
**62**	2age + dis*fly	2age + dis + fly + t	dis + 3age + ct	27	1378.6	17.3
**63**	2age + dis*fly	2age + fly + t	dis + 3age + ct	26	1379.7	16.1
**64**	2age + dis*fly	2age + dis + t	dis + 3age + ct	26	1378.6	15.2

**65**	2age + dis*fly	2age + t	dis + 3age + ct	24	1380.7	12.5
**66**	2age + dis*fly	2age + dis*fly	dis + 3age + ct	18	1391.5	7.8
**67**	2age + dis*fly	2age + fly + dis	dis + 3age + ct	17	1391.8	5.9
**68**	2age + dis*fly	2age + fly	dis + 3age + ct	16	1392.2	4.1
**69**	2age + dis*fly	2age + dis	dis + 3age + ct	16	1392.2	4.1
**70**	2age + dis*fly	2age	dis + 3age + ct	14	1392.7	0.3
**71**	2age + fly + dis	2age + dis*fly + t	dis + 3age + ct	27	1382.5	20.2
**72**	2age + fly + dis	2age + dis + fly + t	dis + 3age + ct	26	1383.0	18.4
**73**	2age + fly + dis	2age + fly + t	dis + 3age + ct	25	1384.3	17.3
**74**	2age + fly + dis	2age + dis + t	dis + 3age + ct	25	1383.0	16.3
**75**	2age + fly + dis	2age + t	dis + 3age + ct	23	1385.3	13.7
**76**	2age + fly + dis	2age + dis*fly	dis + 3age + ct	17	1396.2	9.1
**77**	2age + fly + dis	2age + fly + dis	dis + 3age + ct	16	1396.4	7.2
**78**	2age + fly + dis	2age + fly	dis + 3age + ct	15	1396.7	5.3
**79**	2age + fly + dis	2age + dis	dis + 3age + ct	15	1396.8	5.4
**80**	2age + fly + dis	2age	dis + 3age + ct	13	1397.4	1.7
**81**	2age + fly	2age + dis*fly + t	dis + 3age + ct	26	1394.4	26.7
**82**	2age + fly	2age + dis + fly + t	dis + 3age + ct	25	1394.7	24.9
**83**	2age + fly	2age + fly + t	dis + 3age + ct	24	1403.5	29.1
**84**	2age + fly	2age + dis + t	dis + 3age + ct	24	1394.8	22.7
**85**	2age + fly	2age + t	dis + 3age + ct	22	1404.9	25.9
**86**	2age + fly	2age + dis*fly	dis + 3age + ct	16	1407.1	14.9
**87**	2age + fly	2age + fly + dis	dis + 3age + ct	15	1407.5	13.2
**88**	2age + fly	2age + fly	dis + 3age + ct	14	1415.9	17.2
**89**	2age + fly	2age + dis	dis + 3age + ct	14	1407.5	11.1
**90**	2age + fly	2age	dis + 3age + ct	12	1416.1	13.2
**91**	2age + dis	2age + dis*fly + t	dis + 3age + ct	26	1383.3	18.7
**92**	2age + dis	2age + dis + fly + t	dis + 3age + ct	25	1383.6	16.7
**93**	2age + dis	2age + fly + t	dis + 3age + ct	24	1384.8	15.5
**94**	2age + dis	2age + dis + t	dis + 3age + ct	24	1384.0	14.9
**95**	2age + dis	2age + t	dis + 3age + ct	22	1386.0	12.2
**96**	2age + dis	2age + dis*fly	dis + 3age + ct	16	1396.4	7.1
**97**	2age + dis	2age + fly + dis	dis + 3age + ct	15	1396.5	5.2
**98**	2age + dis	2age + fly	dis + 3age + ct	14	1396.8	3.3
**99**	2age + dis	2age + dis	dis + 3age + ct	14	1397.4	3.8
**100**	**2age + dis**	**2age**	**dis + 3age + ct**	**12**	**1397.9**	**0***
**101**	2age	2age + dis*fly + t	dis + 3age + ct	24	1398.1	25.2
**102**	2age	2age + dis + fly + t	dis + 3age + ct	23	1398.5	23.3
**103**	2age	2age + fly + t	dis + 3age + ct	22	1408.3	28.4
**104**	2age	2age + dis + t	dis + 3age + ct	22	1398.6	21.3
**105**	2age	2age + t	dis + 3age + ct	20	1410.7	25.9
**106**	2age	2age + dis*fly	dis + 3age + ct	14	1410.5	13.3

**107**	*2age*	*2age + fly + dis*	*dis + 3age + ct*	13	1411.0	11.6
**108**	*2age*	*2age + fly*	*dis + 3age + ct*	12	1420.5	16.4
**109**	*2age*	*2age + dis*	*dis + 3age + ct*	12	1411.1	9.6
**110**	*2age*	*2age*	*dis + 3age + ct*	10	1421.2	12.8

Abbreviations: Φ^1= apparent survival first half year Φ^{2+} = apparent survival subsequent full years; *p* = resighting; *K* = number of parameters; *dis*fly* = wintering region; *t* = annual variation; *dis* = distance; *2age* = 2 age classes; *3age* = 3 age classes; *ct* = camera trap; *c* = constant; *fly* = migratory flyway.

Notes: The selected model in step one and step two is highlighted in bold and corresponds to the most parsimonious model with a $\Delta QAIC_c$ < 2.; *$QAIC_c$ = 1040.35; **Model 1 corresponds to the **GOF model which did not distinguish Φ^1 and Φ^{2+}, nor included age effects.**

Table S4. *Reverse stepwise* approach, model selection results by step.

	Model	ϕ^1	ϕ^{2+}	*p*	*K*	Deviance	$\Delta QAIC_c$
**Step 1 - Selection on S**	**1****	*dis*fly * t*		*dis*fly * t*	87	2116.4	690.9
	**2**	*2age * dis*fly * t*	*2age * dis*fly * t*	*dis * 3age * t*	167	1197.2	241.5
	**3**	*2age + dis*fly + t*	*2age + dis*fly + t*	*dis * 3age * t*	86	1297.4	93.2
	**4**	*2age + dis*fly + t*	*2age + dis + fly + t*	*dis * 3age * t*	85	1297.4	90.8
	**5**	*2age + dis*fly + t*	*2age + fly + t*	*dis * 3age * t*	84	1298.2	88.9
	**6**	*2age + dis*fly + t*	*2age + dis + t*	*dis * 3age * t*	84	1297.6	88.5
	**7**	*2age + dis*fly + t*	*2age + t*	*dis * 3age * t*	82	1299.3	84.9
	**8**	*2age + dis*fly + t*	*2age + dis*fly*	*dis * 3age * t*	78	1307.0	80.8
	**9**	*2age + dis*fly + t*	*2age + fly + dis*	*dis * 3age * t*	77	1307.1	78.5
	**10**	*2age + dis*fly + t*	*2age + fly*	*dis * 3age * t*	76	1308.7	77.2
	**11**	*2age + dis*fly + t*	*2age + dis*	*dis * 3age * t*	76	1307.4	76.3
	**12**	*2age + dis*fly + t*	*2age*	*dis * 3age * t*	74	1309.2	72.8
	**13**	*2age + dis + fly + t*	*2age + dis*fly + t*	*dis * 3age * t*	73	1314.2	74.1
	**14**	*2age + dis + fly + t*	*2age + dis + fly + t*	*dis * 3age * t*	84	1302.8	92.3
	**15**	*2age + dis + fly + t*	*2age + fly + t*	*dis * 3age * t*	83	1303.6	90.4
	**16**	*2age + dis + fly + t*	*2age + dis + t*	*dis * 3age * t*	83	1303.0	90.0
	**17**	*2age + dis + fly + t*	*2age + t*	*dis * 3age * t*	81	1304.6	86.3
	**18**	*2age + dis + fly + t*	*2age + dis*fly*	*dis * 3age * t*	77	1317.9	86.3
	**19**	*2age + dis + fly + t*	*2age + fly + dis*	*dis * 3age * t*	76	1311.9	79.6
	**20**	*2age + dis + fly + t*	*2age + fly*	*dis * 3age * t*	75	1313.7	78.5
	**21**	*2age + dis + fly + t*	*2age + dis*	*dis * 3age * t*	75	1312.0	77.2
	**22**	*2age + dis + fly + t*	*2age*	*dis * 3age * t*	73	1314.2	74.1
	**23**	*2age + fly + t*	*2age + dis*fly + t*	*dis * 3age * t*	84	1315.8	101.7
	**24**	*2age + fly + t*	*2age + dis + fly + t*	*dis * 3age * t*	83	1316.6	99.8
	**25**	*2age + fly + t*	*2age + fly + t*	*dis * 3age * t*	82	1325.1	103.6

**26**	*2age + fly + t*	*2age + dis + t*	*dis * 3age * t*	82	1317.3	97.9
**27**	*2age + fly + t*	*2age + t*	*dis * 3age * t*	80	1326.5	99.8
**28**	*2age + fly + t*	*2age + dis*fly*	*dis * 3age * t*	76	1321.3	86.4
**29**	*2age + fly + t*	*2age + fly + dis*	*dis * 3age * t*	75	1321.8	84.3
**30**	*2age + fly + t*	*2age + fly*	*dis * 3age * t*	74	1332.9	90.1
**31**	*2age + fly + t*	*2age + dis*	*dis * 3age * t*	74	1321.8	82.0
**32**	*2age + fly + t*	*2age*	*dis * 3age * t*	72	1333.6	85.8
**33**	*2age + dis + t*	*2age + dis*fly + t*	*dis * 3age * t*	84	1303.7	92.9
**34**	*2age + dis + t*	*2age + dis + fly + t*	*dis * 3age * t*	83	1303.9	90.6
**35**	*2age + dis + t*	*2age + fly + t*	*dis * 3age * t*	82	1304.5	88.6
**36**	*2age + dis + t*	*2age + dis + t*	*dis * 3age * t*	82	1304.8	88.8
**37**	*2age + dis + t*	*2age + t*	*dis * 3age * t*	80	1306.0	84.9
**38**	*2age + dis + t*	*2age + dis*fly*	*dis * 3age * t*	76	1312.1	79.7
**39**	*2age + dis + t*	*2age + fly + dis*	*dis * 3age * t*	75	1312.5	77.6
**40**	*2age + dis + t*	*2age + fly*	*dis * 3age * t*	74	1326.4	85.3
**41**	*2age + dis + t*	*2age + dis*	*dis * 3age * t*	74	1313.2	75.7
**42**	*2age + dis + t*	*2age*	*dis * 3age * t*	72	1315.3	72.5
**43**	*2age + t*	*2age + dis*fly + t*	*dis * 3age * t*	82	1321.3	100.9
**44**	*2age + t*	*2age + dis + fly + t*	*dis * 3age * t*	81	1322.2	99.1
**45**	*2age + t*	*2age + fly + t*	*dis * 3age * t*	80	1331.5	103.4
**46**	*2age + t*	*2age + dis + t*	*dis * 3age * t*	80	1323.4	97.6
**47**	*2age + t*	*2age + t*	*dis * 3age * t*	78	1334.4	100.7
**48**	*2age + t*	*2age + dis*fly*	*dis * 3age * t*	74	1328.8	87.1
**49**	*2age + t*	*2age + fly + dis*	*dis * 3age * t*	73	1329.5	85.2
**50**	*2age + t*	*2age + fly*	*dis * 3age * t*	72	1341.3	91.4
**51**	*2age + t*	*2age + dis*	*dis * 3age * t*	72	1329.8	83.0
**52**	*2age + t*	*2age*	*dis * 3age * t*	70	1342.7	87.6
**53**	*2age + dis*fly*	*2age + dis*fly + t*	*dis * 3age * t*	77	1317.9	86.3
**54**	*2age + dis*fly*	*2age + dis + fly + t*	*dis * 3age * t*	76	1317.9	83.9
**55**	*2age + dis*fly*	*2age + fly + t*	*dis * 3age * t*	75	1318.6	82.0
**56**	*2age + dis*fly*	*2age + dis + t*	*dis * 3age * t*	75	1317.9	81.5
**57**	*2age + dis*fly*	*2age + t*	*dis * 3age * t*	73	1333.3	88.0
**58**	*2age + dis*fly*	*2age + dis*fly*	*dis * 3age * t*	67	1332.1	72.9
**59**	*2age + dis*fly*	*2age + fly + dis*	*dis * 3age * t*	66	1332.3	70.7
**60**	*2age + dis*fly*	*2age + fly*	*dis * 3age * t*	65	1332.7	68.7
**61**	*2age + dis*fly*	*2age + dis*	*dis * 3age * t*	65	1332.5	68.6
**62**	*2age + dis*fly*	*2age*	*dis * 3age * t*	63	1333.0	64.3
**63**	*2age + fly + dis*	*2age + dis*fly + t*	*dis * 3age * t*	76	1321.6	86.6
**64**	*2age + fly + dis*	*2age + dis + fly + t*	*dis * 3age * t*	75	1322.2	84.6
**65**	*2age + fly + dis*	*2age + fly + t*	*dis * 3age * t*	74	1323.1	82.9
**66**	*2age + fly + dis*	*2age + dis + t*	*dis * 3age * t*	74	1322.2	82.3
**67**	*2age + fly + dis*	*2age + t*	*dis * 3age * t*	72	1324.1	78.9

	**68**	*2age + fly + dis*	*2age + dis*fly*	*dis * 3age * t*	66	1337.1	74.2
	**69**	*2age + fly + dis*	*2age + fly + dis*	*dis * 3age * t*	65	1337.4	72.1
	**70**	*2age + fly + dis*	*2age + fly*	*dis * 3age * t*	64	1337.8	70.0
	**71**	*2age + fly + dis*	*2age + dis*	*dis * 3age * t*	64	1337.6	69.9
	**72**	*2age + fly + dis*	*2age*	*dis * 3age * t*	62	1338.1	65.7
	**73**	*2age + fly*	*2age + dis*fly + t*	*dis * 3age * t*	75	1332.5	92.1
	**74**	*2age + fly*	*2age + dis + fly + t*	*dis * 3age * t*	74	1332.8	90.0
	**75**	*2age + fly*	*2age + fly + t*	*dis * 3age * t*	73	1340.2	92.9
	**76**	*2age + fly*	*2age + dis + t*	*dis * 3age * t*	73	1333.0	87.7
	**77**	*2age + fly*	*2age + t*	*dis * 3age * t*	71	1340.4	89.1
	**78**	*2age + fly*	*2age + dis*fly*	*dis * 3age * t*	65	1345.6	78.0
	**79**	*2age + fly*	*2age + fly + dis*	*dis * 3age * t*	64	1346.0	76.0
	**80**	*2age + fly*	*2age + fly*	*dis * 3age * t*	63	1353.3	79.0
	**81**	*2age + fly*	*2age + dis*	*dis * 3age * t*	63	1346.0	73.7
	**82**	*2age + fly*	*2age*	*dis * 3age * t*	61	1353.4	74.5
	**83**	*2age + dis*	*2age + dis*fly + t*	*dis * 3age * t*	75	1322.8	85.1
	**84**	*2age + dis*	*2age + dis + fly + t*	*dis * 3age * t*	74	1323.3	83.0
	**85**	*2age + dis*	*2age + fly + t*	*dis * 3age * t*	73	1323.9	81.1
	**86**	*2age + dis*	*2age + dis + t*	*dis * 3age * t*	73	1323.5	80.8
	**87**	*2age + dis*	*2age + t*	*dis * 3age * t*	71	1325.0	77.2
	**88**	*2age + dis*	*2age + dis*fly*	*dis * 3age * t*	65	1337.4	72.1
	**89**	*2age + dis*	*2age + fly + dis*	*dis * 3age * t*	64	1337.7	70.0
	**90**	*2age + dis*	*2age + fly*	*dis * 3age * t*	63	1337.9	67.8
	**91**	*2age + dis*	*2age + dis*	*dis * 3age * t*	63	1338.3	68.1
	**92**	***2age + dis***	***2age***	*dis * 3age * t*	**61**	**1338.8**	**63.8**
	**93**	*2age*	*2age + dis*fly + t*	*dis * 3age * t*	73	1336.6	90.4
	**94**	*2age*	*2age + dis + fly + t*	*dis * 3age * t*	72	1337.0	88.2
	**95**	*2age*	*2age + fly + t*	*dis * 3age * t*	71	1344.7	91.5
	**96**	*2age*	*2age + dis + t*	*dis * 3age * t*	71	1337.0	85.9
	**97**	*2age*	*2age + t*	*dis * 3age * t*	69	1346.9	88.4
	**98**	*2age*	*2age + dis*fly*	*dis * 3age * t*	63	1348.9	75.8
	**99**	*2age*	*2age + fly + dis*	*dis * 3age * t*	62	1349.1	73.9
	**100**	*2age*	*2age + fly*	*dis * 3age * t*	61	1357.3	77.3
	**101**	*2age*	*2age + dis*	*dis * 3age * t*	61	1349.4	71.6
	**102**	*2age*	*2age*	*dis * 3age * t*	59	1357.8	73.0
**Step 2 - Selection on P**	**103**	*2age + dis*	*2age*	*dis + 3age + t*	18	1390.5	7.1
	**104**	***2age + dis***	***2age***	***dis + 3age + ct***	**12**	**1397.9**	**0.0***
	**105**	*2age + dis*	*2age*	*dis + 2age + ct*	11	1411.4	7.8
	**106**	*2age + dis*	*2age*	*dis + ct*	10	1510.0	77.4
	**107**	*2age + dis*	*2age*	*ct + 3age*	10	1420.7	12.5
	**108**	*2age + dis*	*2age*	*ct*	8	1523.4	83.0
	**109**	*2age + dis*	*2age*	*3age*	9	1457.7	37.3

**110**	*2age + dis*	*2age*	*c*	7	1597.1	134.6

Abbreviations: Φ^1= apparent survival first half year Φ^{2+} = apparent survival subsequent full years; *p* = resighting; *K* = number of parameters; *dis*fly* = wintering region; *t* = annual variation; *dis* = distance; *2age* = 2 age classes; *3age* = 3 age classes; *ct* = camera trap; *c* = constant; *fly* = migratory flyway.

Notes: The selected model in step one and step two is highlighted in bold and corresponds to the most parsimonious model with a $\Delta QAIC_c$ < 2. *$QAIC_c$ = 1040.35; **Model 1 corresponds to the **GOF model which did not distinguish Φ^1 and Φ^{2+}, nor included age effects.**
